# Interpapillary muscle distance independently predicts recurrent mitral regurgitation

**DOI:** 10.1186/s13019-024-02631-z

**Published:** 2024-03-20

**Authors:** Ivancarmine Gambardella, Cristiano Spadaccio, Sanjeet S. A. Singh, Yasushige Shingu, Takashi Kunihara, Satoru Wakasa, Francesco Nappi

**Affiliations:** 1 Department of Cardiothoracic Surgery, Weill Cornell Medicine, New York, USA; 2 Department of Cardiothoracic Surgery, Mayo Clinic, Rochester, USA; 3 Department of Cardiothoracic Surgery, Royal Infirmary of Edinburgh, Edinburgh, UK; 4 Department of Cardiovascular Surgery, Faculty of Medicine and Graduate School of Medicine, Hokkaido University, Sapporo, Japan; 5 Department of Cardiac Surgery, The Jikei University School of Medicine, Tokyo, Japan; 6 Department of Cardiac Surgery, Centre Cardiologique du Nord, Saint-Denis, France

**Keywords:** Ischaemic secondary mitral regurgitation, Papillary muscle approximation, Restrictive mitral annuloplasty, Interpapillary muscle distance

## Abstract

**Objective:**

Ischaemic secondary mitral regurgitation (ISMR) after surgery is due to the displacement of papillary muscles resulting from progressive enlargement of the left ventricle end-diastolic diameter (LVEDD). Our aim was to prove that if the interpapillary muscle distance (IPMD) is surgically stabilized, an increase in LVEDD will not lead to a recurrence of ischaemic mitral regurgitation (MR).

**Methods:**

Ninety-six patients with ISMR, who underwent surgical revascularisation and annuloplasty, were randomly assigned in a 1:1 ratio to undergo papillary muscle approximation (PMA). At the 5-year follow-up, we assessed the correlation between PMA and echocardiographic improvements, the effect size of PMA on echocardiographic improvements, and a prediction model for recurrent MR using inferential tree analysis.

**Results:**

There was a significant correlation between PMA and enhancements in both the α and β angles (Spearman’s rho > 0.7, *p* < 0.01). The α angle represents the angle between the annular plane and either the A2 annular-coaptation line or the P2 annular-coaptation line. The β angle indicates the angle between the annular plane and either the A2 annular-leaflet tip line or the P2 annular-leaflet tip line. PMA led to substantial improvements in LVEDD, tenting area, α and β angles, with a large effect size (Hedge’s g ≥ 8, 95% CI ORs ≠ 1). The most reliable predictor of recurrent MR grade was the interpapillary distance, as only patients with an interpapillary distance greater than 40 mm developed ≥ 3 + grade MR. For patients with an IPMD of 40 mm or less, the best predictor of recurrent MR grade was LVEDD. Among the patients, only those with LVEDD greater than 62 mm showed moderate (2+) MR, while only those with LVEDD less than or equal to 62 mm had absent to mild (1+) MR.

**Conclusion:**

Prediction of recurrent ischaemic MR is not independent of progressive LVEDD increase. PMA-based surgical procedure stabilises IPMD.

**Supplementary Information:**

The online version contains supplementary material available at 10.1186/s13019-024-02631-z.

## Introduction

The management of secondary ischemic mitral regurgitation (SIMR) is a topic of intense debate in the field of cardiac surgery. However, there is one aspect that is indisputable: the etiopathology of ischemic MR is predominantly caused by chordal tethering of the valvular leaflets, resulting from displacement of the papillary muscles. This displacement is a consequence of the gradual enlargement of the ischemic left ventricle [1]. Apparently, complete revascularisation of the ischemic myocardial segments could potentially resolve ischemic MR, assuming that the myocardial segments remain viable. This is often the scenario for moderate ischemic MR, which is likely due to moderate chronic myocardial ischemia. In this group of patients, only coronary revascularization is needed to satisfactorily resolve the MR without requiring additional procedures. However, such a simple approach is not suitable for higher degrees of MR, which are conceivably the result of more severe myocardial damage. This specific patient group is prone to exhibiting a coronary disease pattern that opposes full revascularisation and non-viable myocardial segments. This results in a progressive eccentric hypertrophy of the impaired ventricle, with gradual papillary muscle displacement and chordal tethering. In this situation, it is customary to supplement coronary revascularisation with a mitral procedure [1]. There is a significant inconsistency between observational and clinical experimental data regarding the best procedure for mitral treatment in this population subset. This subset of patients is more likely to present with a pattern of coronary disease that is hostile to complete revascularization, and myocardial segments that are not viable. The consequence is progressive eccentric hypertrophy of the impaired ventricle, with a gradual displacement of the papillary muscles alongside chordal tethering. For this subset of patients, the addition of a mitral procedure to coronary revascularization is customary [1]. Our group's latest meta-analysis focused on the most relevant data currently available (i.e. randomized controlled trials [RCT] and propensity-score matched cohorts only). We found that mitral valve replacement (MVR) increased operative mortality, but resulted in decreased recurrent MR and reoperation rates during follow-up when compared to mitral restrictive annuloplasty [2]. The final element of the evidence jigsaw is created by combining restrictive mitral annuloplasty (RMA) with papillary muscle approximation (PMA). In our group's PMA RCT-Analysis study [3], we compared RMA to PMA alongside RMA for treating severe ischemic MR (with complete revascularisation received by both groups). The inclusion of PMA led to favourable ventricular remodelling and performance 5 years later: left ventricle end-diastolic diameter (LVEDD) experienced an average decline of − 5.8 ± 4.1 mm compared to − 0.2 ± 2.3 mm in the group without PMA (*p* < 0.01); and left ventricular ejection fraction (LVEF) had an average increase of 8.8 ± 5.9% compared to 2.5 ± 4.3% in the group without PMA (*p* < 0.01). Over the 5-year period, the occurrence of moderate-severe MR reoccurred in 4.7% compared to 22.9% in those who had and did not have PMA, respectively (*p* = 0.01) [3]. However, there is limited understanding regarding the physiological basis behind the advantage of reapproximating the papillary muscles through surgery and whether particular effects of this procedure on the geometry or conformation of the mitral apparatus are responsible for its positive impact on adverse left ventricular remodeling and recurrence of mitral regurgitation.

With this secondary analysis of the PMA trial, our aim is to establish a predictive model that identifies the correlation between post-PMA parametric improvements and their impact on mitigating the effect of progressive LVEDD enlargement on recurrent ischemic MR in a hierarchical manner.

## Methods

### Study design

The initial PMA RCT was included, with an identification number of 202201143 from the institutional review board (IRB), in the Transcatheter Edge to Edge Mitral Valve Repair vs Standard Surgical Mitral Valve Operation for Secondary Mitral Valve Regurgitation project (TEERMISO). TEERMISO is a prospective comparison of surgical and percutaneous interventions for secondary MR. The TEERMISO protocol was registered in advance with identification number NCT05090540 on the Protocol Registration and Result System of ClinicalTrials.gov, along with declaration of conformity number 2222269v0. TEERMISO is recruiting from several centres, which include Hokkaido University Hospital in Japan, Mondor University Hospital in France, Biomedical Campus in Italy, Centre Cardiologique du Nord in France, and Aberdeen Royal Infirmary in the United Kingdom. Enrollment for TEERMISO will cease in December 2023, with overall results to be produced thereafter. The PMA RCT was a clinical trial that randomly selected patients with severe chronic ischemic MR. The patients were divided into two groups: those who underwent isolated RMA and those who underwent RMA plus PMA (the latter group will be referred to as the "PMA group" for simplicity). Both groups received complete coronary artery bypass grafting (CABG). The trial was conducted in a prospective manner. The study was originally planned to identify a 4 mm reduction in LVEDD at the end of the trial compared to the baseline (1 vs. 5 mm) between the groups, with an equal allocation ratio of 1:1, a standard deviation of 5 mm, a 5% α-error, and 90% power. The initial sample size calculation indicated 35 patients per group for follow-up. However, this number was raised to 48 per group to accommodate unexpected variability and potential loss of one-third of patients during the follow-up period. Patient selection for the PMA RCT took place in three central Italian regions (Lazio, Campania, Abruzzo), with all participants referred to the Bio-Medical Campus academic tertiary centre in Rome.

Enrolment started in May 2007 and finished in November 2010. The surgical procedures were carried out by three different surgeons, and the follow-up concluded in November 2015.

Patient consent was obtained after the assigned IRB approval number 202201173 in accordance with the research guidance. This study complies with the Declaration of Helsinki.

### Eligibility

Adults with coronary artery disease affecting multiple arteries and moderate to severe ischemic mitral regurgitation were eligible to take part in the study. Severe mitral regurgitation (MR) was classified as an effective regurgitant orifice area (EROA) of at least 0.4 cm^2^ or greater, identified via transthoracic echocardiography (TTE). Eligible patients had coronary targets that were affected by proximal lesions of high grade, which compromise viable myocardium that is ischemic. Exclusion criteria comprised any echocardiographic evidence of structural mitral valve pathology, myxomatous disease, prior endocarditis, rheumatic valve disease, leaflet prolapse, additional planned valvular or aortic concomitant procedure (e.g. surgery on tricuspid valve or thoracic aorta), congenital heart disease, earlier cardiac surgery, pre-operative inotrope support or mechanical circulatory assistance, or chronic renal replacement therapy. Chronic renal insufficiency is defined as having creatinine ≥ 2. 5 mg/dL or chronic renal replacement therapy; hepatic cirrhosis or synthetic failure; pregnancy at the time of randomization; simultaneous disease limiting life expectancy to less than 2 years.

### Randomization

All enrolled patients provided written informed consent for the study and surgical procedure. Randomization was performed centrally and intra-operatively after sternotomy but before cannulation of the aorta to reduce the probability of including patients with unexpected surgical contraindications for mitral valve repair. After conducting checks, the treatment assignment was sent to the study coordinator and the intervention was carried out. Therefore, the primary efficacy analysis was conducted using the intention-to-treat approach.

### Interventions

The standard RMA was supplemented with the standardized subvalvular repair, involving the reapproximation of both papillary muscles (PMs), to achieve correction of MR and restoration of the subvalvular apparatus using the following procedure:

1. Reposition of the PMs. (Additional file 1: Fig. 1).
2. The realignment of the subvalvular apparatus in one plane in a standardised manner. both PMs to eliminate apical tenting of the anterior leaflet (Additional file 1: Fig. 2).
3. For PMs with anatomy types 1–3, both PMs are approximated using Goretex 4–0 stitch (CV-4, W.L. Gore & Associates, Newark, Delaware, USA). For PMs with anatomy types 4–5, a 4 mm Goretex graft (Gore & Associates, Newark, Delaware, USA) is used for approximation through the sling procedure (Additional file 1: Fig. 1).

**Fig. 1:**
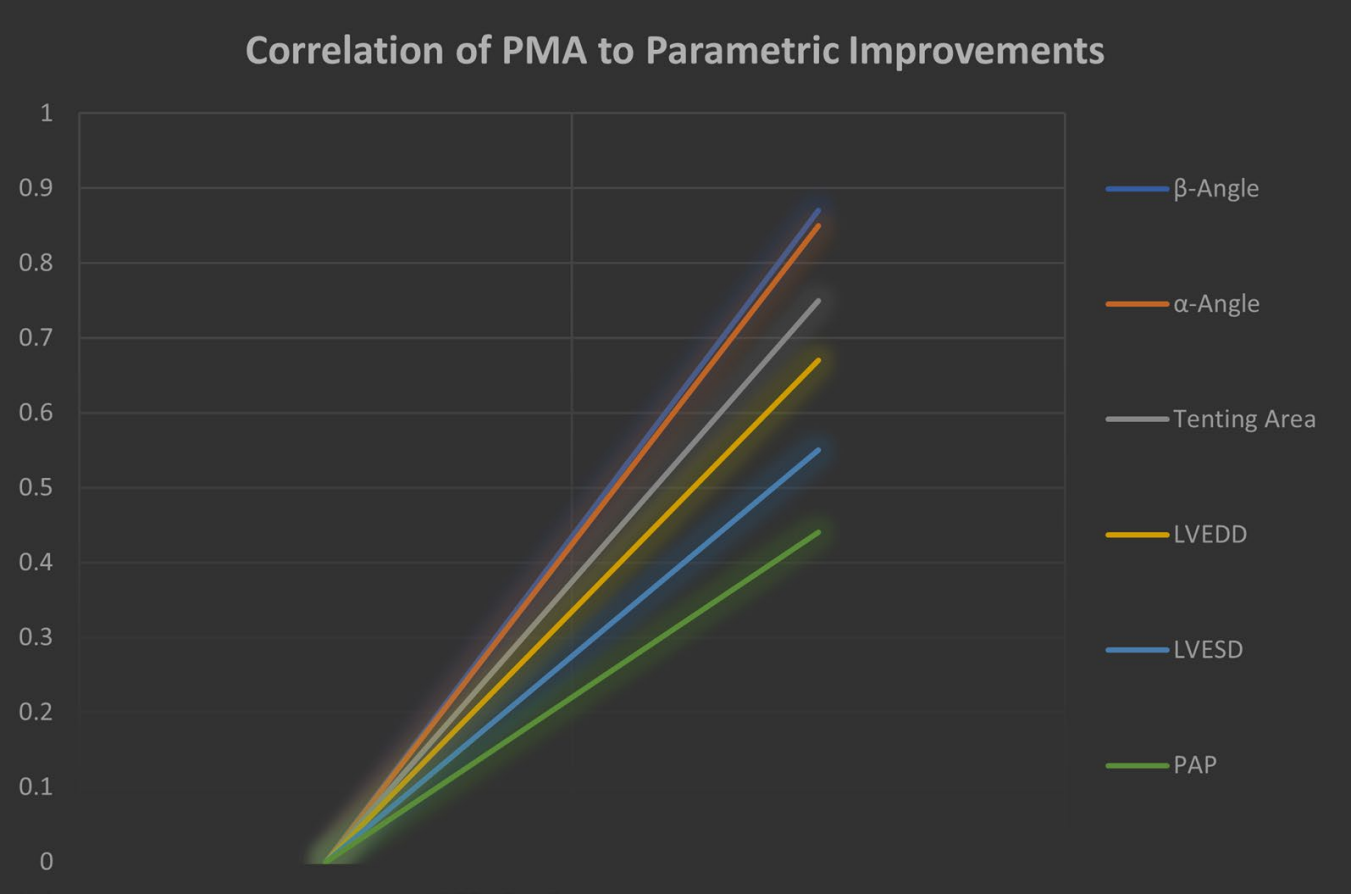
Correlation between papillary muscle approximation to parametric improvements. A parametric improvement was defined as a beneficial Δ between preoperative and 5-year postoperative values.The correlation magnitude was evaluated with Spearman’s rho. The magnitude of correlation was high for improvements of the α and β angles, and of the tenting area. The magnitude of correlation was moderate for improvements of the LVEDD and LVESD.The magnitude of correlation was low for improvements of the PAP. Acronyms: LVEDD = left ventricular end diastolic volume; LVESD = left ventricle end systolic diameter; PAP = (systolic) pulmonary artery pressure

**Fig. 2:**
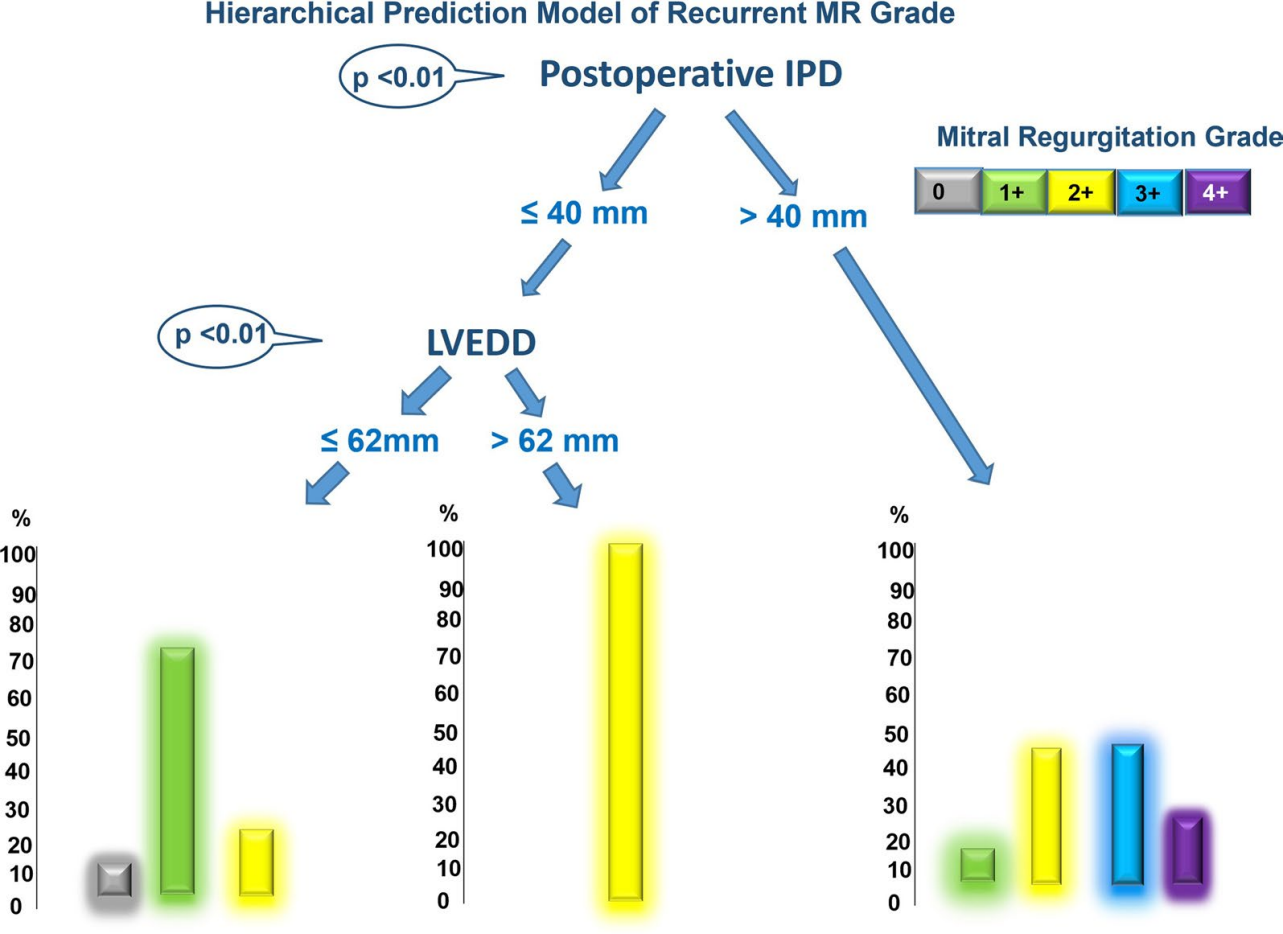
Hierarchical model to predict the degree of recurrent mitral regurgitation (MR), with the use of the machine learning algorithm of inferential tree analysis.Interpapillary distance was the best predictor of recurrent MR grade. Only patients with an interpapillary distance > 40 mm developed 3–4 + recurrent MR. In patients with an interpapillary distance ≤ 40 mm, the left ventricle end diastolic diameter was the best predictor of recurrent MR grade. Indeed, only patients with a diameter > 62 mm developed 2 + recurrent MR, and only patients with a diameter ≤ 62 mm had absent to mild recurrent MR

In the parasternal short-axis view of the LV, end-diastolic and end-systolic interpapillary muscle distances (IPMD) were compared with preoperative values measured by transthoracic echocardiography. Comparing IPMD in physiological and pathological conditions is crucial for the procedure's success. In normal conditions, the distance between the interpapillary muscles measured in mid-systole is between 12.9 and 22.5 mm. It should be noted that these measurements exhibit variability when adjusted for BSA and age. The BSA- and age-normalized value for IPMD is approximately 10.5 mm plus or minus 3.3 mm. The mean decrease in the interpapillary distance was 30%, with a subsequent decrease observed in the subsequent 6 months post-surgery as a result of the advantageous reshaping of the left ventricle.

Coronary artery bypass surgery was conducted employing standard surgical approaches to achieve complete revascularisation using single or double internal thoracic artery (ITA) grafting. All patients were administered guideline-directed medical therapy to manage heart failure and coronary artery disease which included antiplatelet medication, neurohormonal antagonists, lipid-lowering agents, and appropriate cardiac resynchronization and defibrillator therapy. Patients were monitored for up to 5 years post-surgery. Details of the surgical procedure are in the supplementary material.

### Endpoints of the secondary analysis

We established that a beneficial change occurred if there was a positive difference between the measurement of a specific echocardiographic parameter before the surgery and 5 years after. The parameters evaluated include the α-angle (tilt angle of the front leaflet), β-angle (tilt angle of the back leaflet), tenting area, interpapillary muscle distance (IPMD), LVEDD, left ventricle end-systolic diameter (LVESD), LVEF, and pulmonary artery systolic pressure (PAP). The main objective was to create a hierarchical model that forecasts the extent of recurring ischemic MR following surgical repair. As secondary objectives, we assessed the correlation of PMA with parametric improvements and the effect size of PMA on parametric improvements.

### Statistical analysis

The correlation between PMA and improvements in parameters was analysed using Spearman’s rho. We defined the magnitude of correlation as low (rho 0–0.49), moderate (rho 0.50–0.69), or high (rho 0.70–1).

To evaluate the association between the differences in echocardiographic parameter measurements taken before surgery and 5 years after, and recurrent MR, we used logistic regression. All variables that demonstrated a significance level of *p* ≤ 0.2 in univariable regression were incorporated into the multivariable model.

A decision tree algorithm was used to identify the relationship between dependent and independent variables through recursive partitioning. If the null hypothesis of independence was rejected, the independent variable with the strongest correlation to the dependent variable was chosen. The data was then partitioned according to the selected independent variable and the process was repeated. The Strasser and Weber permutation test was used for conditional inference [4, 5]. Consistency was maintained with metrics and units. Fillers were removed for conciseness.

The statistical analyses were conducted using R Studio with the following packages: `stats’, `sjPlot’, `car’, `effectsize’, `ggplot2’, `ggfortify’, `tidyr’, `dplyr’, `binda’, `tidyverse’, `ctree’, `rpart’, and `rpart.plot’.

## Results

### Preoperative and operative variables

There were no differences between the RMA and PMA groups in terms of demographics, baseline characteristics, medications, and cardiac status before the operation (*p* > 0.05 for all) (Table 1). Moreover, the operative variables did not differ between the two groups (*p* > 0.05 for all) (Table 2). Operative mortality occurred in 7 patients (4 in the RA and 3 in the PMA groups). At 5-year follow-up, the cumulative rate of mortality was similar between groups (RA 29.2% vs. PMA 22.9%, *p* = 0.50).

**Table 1:** Preoperative variables of 96 patients undergoing surgical repair for ischemic mitral valve regurgitation. All patients received complete surgical coronary revascularization and restrictive mitral annuloplasty (RMA).After 1:1 randomization, 48 patients also received papillary muscle approximation (PMA).CCS = Canadian Cardiovascular Society

Preoperative variables			
	RA—N^o^ 48	PMA – N^o^ 48	*P* value
*Baseline characteristics*			
Male sex	30 (62.5)	28 (58.3)	0.676
Age	64.6 ± 7.4	62.9 ± 7.0	0.310
Medical history			
Hypertension	23 (47.9)	23 (47.9)	1.000
Dyslipidemia	20 (41.7)	18 (37.5)	0.676
Diabetes	20 (41.7)	18 (37.5)	0.676
Chronic kidney disease stage III	11 (22.9)	12 (25.0)	0.811
Familiarity for cardiovascular disease	12 (25.0)	12 (25.0)	1.000
Chronic obstructive pulmonary disease	6 (12.5)	7 (14.6)	0.827
Atrial fibrillation	10 (20.8)	9 (18.7)	0.798
Heart Failure	36 (75.0)	33 (68.7)	0.496
*Preoperative medications*			
Beta-blockers	40 (83.3)	41 (85.4)	0.779
Calcium-channel blockers, dihydropyridines	23 (48.9)	26 (54.2)	0.610
Nitrates	31 (64.6)	29 (60.4)	0.673
Angiotensin converting enzyme inhibitors	40 (83.3)	39 (81.2)	0.789
Angiotensin receptor blockers	8 (16.7)	9 (18.7)	0.789
Diuretics	48 (100.0)	48 (100.0)	1.000
*Cardiac status*			
Previous myocardial infarction			0.948
Inferior	29 (60.4)	29 (60.4)	
Antero-infero-posterior	10 (20.8)	11 (22.9)	
Antero-lateral	9 (18.7)	8 (16.7)	
Grade on CCS angina scale—n%			0.834
No angina	29 (60.4)	30 (62.5)	
Grade III or IV	19 (39.6)	18 (37.5)	
Coronary angiography: diseased vessels (stenosis > 60%)			
Left anterior descending	30 (62.5)	31 (64.6)	0.832
Left circumflex	32 (66.7)	34 (70.8)	0.660
Right coronary artery	40 (83.3)	42 (87.5)	0.562
Surgery within 60 days from infarction	40 (83.3)	42 (87.5)	0.563
New York Heart Association functional class			0.496
3	12 (25.0)	15 (31.2)	
4	36 (75.0)	33 (68.7)	
Minnesota Living with Heart Failure score	42.6 ± 12.4	43.7 ± 12.7	0.669

**Table 2:** Operative variables of 96 patients undergoing surgical repair for ischemic mitral valve regurgitation.All patients received coronary artery bypass grafting (CABG) and reduction annuloplasty (RA).After 1:1 randomization, 48 patients also received papillary muscle approximation (PMA).ACX = aortic cross clamp time; CPB = cardiopulmonary bypass time

Operative variables			
	RA—N^o^ 48	PMA—N^o^ 48	*P* value
Undersizing annuloplasty, n%	48 (100)	48 (100)	1.000
Ring size used			0.536
Physio Annuloplasty Ring N° 26 n%	19 (39.6)	22 (45.8)	
Physio Annuloplasty Ring N° 28 n%	29 (60.4)	26 (54.2)	
CABG, n	48 (100)	48 (100)	1.000
Number of grafts			0.643
1	12 (25.0)	12 (25.0)	
2	4 (8.3)	2 (4.2)	
3	17 (35.4)	14 (29.2)	
4	15 (31.2)	20 (41.7)	
mean	2.7 ± 1.2	2.9 ± 1.2	0.549
Internal mammary artery			0.904
0	4 (8.3)	4(8.3)	
1	16 (33.3)	14(29.2)	
2	28 (58.3)	30(62.5)	
AXC time, min	93.4 ± 6.3	100.8 ± 9.9	< 0.001
CPB time, min	108.1 ± 8.4	116.3 ± 9.2	< 0.001

### Correlation of PMA with parametric improvements

A comparison of echocardiographic parameters preoperatively, at 1-year and 5-years follow-up is detailed in Table 3. The addition of PMA led to a significant improvement in all parameters for 5 years post-surgery (p < 0.01 for all). The improvement in the tethering angles (α-angle: rho 0.85; β-angle: rho 0.87) and tenting area (rho 0.75) correlated strongly with the addition of PMA. The ventricular diameters (LVEDD: rho 0.67; LVESD: rho 0.55) also improved moderately. The addition of PMA had a low correlation with improvements in PAP (rho 0.44) (Fig. 1).

**Table 3:** Pre-operative, 1-year and 5-year postoperative echocardiographic parameters in patients with ≥ 3 ischemic mitral regurgitation, undergoing coronary artery bypass grafting + restrictive annuloplasty (RA) vs papillary muscle approximation (PMA). Abbreviations and acronyms: IPD, interpapillary muscle distance; LVEF, left ventricular ejection fraction; MR, mitral regurgitation; LVEDD, left ventricular end diastolic diameter; LVESD, left ventricular end systolic diameter; PASP, pulmonary artery systolic pressure

Parameter	Pre-Operative		1-Year Follow-Up		5-Yeas Follow-Up	
	RA—N^o^48	PMA—N^o^48	RA—N^o^38	PMA—N^o^40	RA—N^o^ 26	PMA—N^o^37
*α-Angle, *^*0*^*—mean (SD)*	31.6 (2.2)	31.5 (2.3)	31.1 (1.6)	21.3 (3.1)	31.8 (2.0)	21.9 (2.8)
*β-Angle, *^*0*^*—mean (SD)*	54.1 (5.6)	54.5 (5.0)	42.5 (6.3)	38.2 (2.8)	46.4 (9.9)	38.3 (2.9)
*Tenting Area, mm*^*2*^*—mean (SD)*	2.8 (0.3)	2.9 (0.3)	0.9 (0.1)	0.8 (0.2)	2.02 (0.3)	1.0 (0.3)
*Tenting Height, mm—mean (SD)*	1.2 (0.2)	1.2 (0.1)	0.7 (0.2)	0.6 (0.1)	0.8 (0.1)	0.7 (0.2)
*LVEDD, mm—mean (SD)*	61.4 (3.7)	62.7 (3.4)	55.4 (3.1)	55.9 (3.1)	60.6 (4.6)	56.4 (5.7)
*LVESD, mm—mean (SD)*	52.2 (3.4)	53.4 (3.5)	46.2 (3.0)	46.9 (2.6)	50.2 (4.3)	47.1 (5.9)
*ES-IPMD, mm—mean (SD)*	44.2 (3.9)	44.7 (4.2)	35.6 (6.1)	37.2 (2.7)	37.4 (5.0)	31.9 (4.1)
*PASP, mmHg—mean (SD)*	47.9 (2.8)	48.8 (5.0)	43.1 (3.0)	41.8 (3.1)	44.5 (2.9)	44.8 (3.5)
≥ *3 MR—n (%)*	48/48 (100)	48/48 (100)	0/38 (0.0%)	0/40 (0.0%)	9/26 (26.5%)	1/37 (2.7%)
*LVEF, %—mean (SD)*	36.7 (3.7)	35.0 (5.3)	42.5 (2.3)	42.9 (5.8)	39.9 (3.9)	44.1 (6.0)

Legend: Pre-operative, 1-year and 5-year postoperative echocardiographic parameters in patients with ≥ 3 ischemic mitral regurgitation, undergoing coronary artery bypass grafting + restrictive annuloplasty (RA) vs papillary muscle approximation (PMA). Abbreviations and acronyms: ES-IMPD,  end systole interpapillary muscle distance; LVEF, left ventricular ejection fraction; MR,  mitral regurgitation; LVEDD,  left ventricular end diastolic diameter; LVESD,  left ventricular end systolic diameter; PASP,  pulmonary artery systolic pressure

### Independent association of PMA to parametric improvements

Recurrent MR was found to be associated with the differences (Δs) between preoperative and 5-year postoperative values in all investigated parameters (Table 3) during univariable analysis. During multivariable analysis, the IPMD’s Δ was identified as the only independent factor related to the recurrence of 3–4 + MR with a significant effect size (odds ratio (OR) 2.55, 95% confidence interval (CI) 1.01 | 7.24). The difference of the tenting area was linked to recurrent MR independently, albeit with a reduced impact (OR 0.28, 95% CI 0.05 | 0.88) (Table 4).

**Table 4:** The Δs (differences between preoperative and 5 years postoperative values) in the measurement of echocardiographic parameters were evaluated individually (univariate regression) and jointly (multivariable regression) as predictor of recurrent mitral valve regurgitation. Abbreviations and acronyms: IPD = interpapillary distance;LVEDD = left ventricular end diastolic diameter;LVESD = left ventricular end systolic diameter

Predictors of Recurrent ≥ 3 + Mitral Regurgitation						
Predictor	Univariate			Multivariable		
	OR	95% CI	P value	OR	95% CI	*P* value
Δ of α-Angle	1.13	1.03 | 1.25	< 0.01	1.1	0.78 | 1.50	0.55
Δ of β-Angle	1.03	1.01 | 1.05	< 0.01	1	0.94 | 1.09	0.88
Δ of Tenting Area	2.01	1.06 | 4.03	0.04	0.28	0.05 | 0.88	0.40
Δ of LVESD	1.43	1.20 | 1.77	< 0.01	1.44	0.98 | 2.27	0.07
Δ of LVEDD	1.33	1.16 | 1.57	< 0.01	1.1	0.76 | 1.62	0.6
Δ of IPMD	2.69	1.24 | 6.34	0.01	2.55	1.01 | 7.24	0.05

Legend: The Δs (differences between preoperative and 5 years postoperative values) in the measurement of echocardiographic parameters were evaluated individually (univariate regression) and jointly (multivariable regression) as predictor of recurrent mitral valve regurgitation. Abbreviations and acronyms: IPD,  interpapillary distance; LVEDD, left ventricular end diastolic diameter; LVESD,  left ventricular end systolic diameter

### Best predictors of recurrent IMR

The postoperative IPMD was found to be the most reliable indicator of recurrent MR severity. Patients with an IPMD > 40 mm experienced ≥ 3 + grade MR (11/20, 55%, *p* < 0.01). In patients with an IPMD ≤ 40 mm, a patient's LVEDD was the best predictor of the grade of recurrent MR.

Specifically, moderate (2+) MR was only present in patients with an LVEDD > 62 mm, while absent to mild (1+) MR was limited to patients with LVEDD ≤ 62 mm (as shown in Fig. 2).

## Discussion

The initial analysis of the PMA randomised clinical trial's findings improves our understanding of the benefits of using PMA with RMA compared to using RMA alone for treating severe ischemic mitral regurgitation. The 5-year results indicate that the combination of papillary muscle surgery and annuloplasty was more effective than standard annuloplasty alone for the treatment of ischemic MR. This resulted in improved ventricular geometry, remodeling, and function. The research uncovered a significant difference between groups in the rank-based evaluation of left ventricular reverse remodeling after 5 years. The findings demonstrated a consistent and substantial enhancement in LVEDD, LV end-systolic diameter, and ejection fraction in the PMA cohort. On the other hand, the RMA alone group displayed progressive ventricular cavity enlargement and LV function decline, indicating inadequate counteraction or prevention of detrimental remodeling that occurred over time in ischemic MR [3].

In this second analysis, our objective is to thoroughly examine how reducing interpapillary distance through surgical approximation of papillary muscles can recover left ventricular geometry whilst mitigating the risk of recurrent MR.

### Principal findings

The reappearance of ischemic MR after RMA ± CABG is traditionally thought to occur due to progressive LVEDD enlargement caused by chordal tethering. However, our findings illustrate that progressive LVEDD enlargement does not lead to the recurrence of ischemic MR, as long as the IPMD is surgically stabilized by adding PMA. Possibly, the combination of RMA and PMA provides a solution for both the valvular and sub-valvular mitral apparatus, similarly to MVR with chordal sparing.

### PMA affects normal spatial relationships of the components of MV in patients with SIMR

The erroneous assumption that the leaflet tethering in ischemic MR resulted from apical displacement of the papillary muscles was initially made due to the use of tethering lengths (2D parameters) in earlier studies to evaluate the 3D displacement of the muscles [6–14]. However, subsequent studies looking at the effect of LVEDD enlargement in ischemic MR with a 3D assessment challenged this notion. Experimental induction of acute ischemic MR did not displace the papillary muscles at the apex. Instead, it restricted the distance between the posterior commissure and the posterior papillary muscle, according to reports [15–17]. Moreover, three-dimensional evidence reveals that experimental models studying non-acute ischemic MR yield fitting results. In cases of sub-acute or chronic ischemic MR, an enlarged LVEDD results in decreased mitral annulus to posterior papillary muscle distance compared to the control group (3.16 ± 0.41 cm vs. 3.82 ± 0.53 cm respectively, p = 0.01). This change in shape resulted from two different forces affecting the posterior papillary muscle—a force towards the back (0.58 ± 0.4 cm compared to 1.39 ± 0.46 cm in the control and ischemic groups, p < 0.01) and a force from the side (0.66 ± 0.55 cm compared to 1.19 ± 0.70 cm in the control and ischemic groups, *p* < 0.01). On the other hand, the ischemic enlargement of the LVEDD did not significantly shift the anterior papillary muscle. Hence, 3D data contradicts the idea that ischemic MR is caused by displacement of the apical papillary muscle and points instead to displacement of the posterior papillary muscle in the posterolateral region as the primary cause. This hypothesis is supported by studies that have attempted to rectify the sub-valvular vectors. Balloon repositioning of a posterior papillary muscle, which was displaced, along an anteroseptal vector resulted in reduced tethering and MR [18, 19]. The Coapsys device (Myocor Inc, Maple Grove, Minnesota), which redirects the posterior papillary muscle with an anteroseptal vector, yielded comparable results [20]. Additionally, repositioning the posterior papillary muscle towards the right fibrous trigone (anteroseptal vector) decreased leaflet tethering [21]. Conversely, pulling the posterior papillary muscle towards the posterior commissure was unsuccessful in correcting ischemic MR as confirmed by the evidence [22].

RMA worsens posterolateral tethering by pulling the annulus further from the displaced posterior papillary muscle [23]. This can result in residual and recurrent regurgitation [24]. Numerous surgical groups have published clinical evidence that supports these experimental findings. For instance, our PMA trial's primary analysis revealed that PMA decreases recurrent MR by moving the posterior papillary muscle towards the anterior septum instead of the ventricular base [3, 25]. Our findings align with those of other surgical groups [26–31].

Most clinicians prefer valve replacement over repair to treat ischemic mitral regurgitation based on the results of the CTSN trial [24, 32]. However, the trial's comparison of MVR to RMA alone (without PMA) is insufficient in this context, as demonstrated. Therefore, a trial comparing MVR versus RMA combined with PMA would be advantageous.

The optimal treatment for ISMR remains a topic of debate. Our analysis underscores the need to effectively address both the valvular and subvalvular components of the mitral valve to prevent the recurrence of mitral regurgitation, which results from sustained ventricular geometrical distortion. Limiting the annular diameter or solely focusing on the leaflets through procedures like transcatheter therapies [31–34] may prove inadequate in attaining LV geometry restoration and preventing adverse remodeling consequences.

Currently, no published or ongoing trials have been developed to evaluate the clinical impact of the modulation of the IPMD in ISMR [33–39]. The present and previous studies from other groups appear to endorse the theory that restoring the functional spatial relationships between the various components of the mitral valve apparatus is vital in ensuring long-lasting outcomes post-surgery [3, 25–31].

Repairing the mitral valve using valvular techniques to avoid future surgeries is crucial in patients with small preoperative ventricular dimensions during standard surgical procedures [24]. Additionally, individuals who have undergone transcatheter edge-to-edge repair (TEER) should receive RMA [33] .

The CTSN trial showed that RMA provides clear benefits for patients with smaller left ventricular size [24]. Out of the 74 patients with severe ISMR who underwent the RMA procedure and did not have persistent or recurring mitral regurgitation, their left ventricle measured significantly smaller (43 ± 26 mL/m^2^) 2 years post-procedure as compared to those who had recurring mitral regurgitation following RMA (63 ± 27 mL/m^2^). Moreover, their left ventricle was smaller than anticipated in comparison to patients who had gone through MVR (61 ± 39 mL/m^2^) [24].

Hamasi et al. [33] investigated the prognostic significance of altered mitral valve geometry in patients receiving TEER or medical therapy (GDMT). In heart failure patients with severe secondary mitral regurgitation, a significant anteroposterior mitral annular diameter and higher effective regurgitatant orifice area (EROA) proved to be strong echocardiographic indicators for heart failure hospitalization and mortality risk. These findings are applicable to patients who have received treatment with GDMT alone or with the additional TEER procedure. The Alfieri stitch does not prevent recurrence of MR regurgitation, highlighting the need to treat an increased anteroposterior diameter with RMA.

The significance of papillary muscles was confirmed by the Osaka group’s study [40]. Kainuma et al. discovered that resolution of leaflet tethering cannot be achieved by RMA alone and that the IPMD is primarily responsible for the reappearance of mitral regurgitation. Left ventricle reverse remodelling conducted after RMA reduced the IPMD from 31 ± 6 to 25 ± 5 mm, thereby potentially compensating for the greater angle of the posterior leaflet and augmenting its advantages [40].

### Limitations

This research study encompasses all limitations outlined for the primary analysis [3], alongside supplementary limitations that are characteristic of secondary analyses [41]. The authors also acknowledge that the evidence supporting PMA as an adjunct to RMA is currently not strong, being backed only by one RCT and a few observational analyses. It is necessary to conduct further RCTs to establish PMA as a recommended procedure.

## Conclusion

During the research, surgery of the papillary muscles for SIMR led to significant enhancements in the left ventricle's geometry and distortion, ultimately restoring the normal spatial relationships between all parts of the mitral valve. This procedure has effectively lowered the risk of mitral regurgitation recurrence. Furthermore, it was discovered that surgical stabilization of the IPMD can decrease the connection between the gradual rise in the LVEDD and recurring ischemic MR. Further randomised multicentre clinical trials with a minimum follow-up of 5 years are required to directly compare patients undergoing either PMA alongside RMA and MVR, or transcatheter edge-to-edge repair, for the management of SIMR.

### Supplementary Information

**Additional file 1: Supplementary Figure 1.** Combined papillary muscle approximation and restrictive mitral annuloplasty.** Supplementary Figure 2.** The anatomy of the PMs (papillary muscles) is the key to surgical correction.

## Data Availability

Drs Nappi, Gambardella, had full access to all of the data in the study and take responsibility for the integrity of the data and the accuracy of the data analysis. The data underlying this article will be shared on reasonable request to the corresponding author.
