# Supplementary material for: Interpapillary muscle distance independently predicts recurrent mitral regurgitation
Source: J Cardiothorac Surg. 2024 Mar 20;19:147. doi: 10.1186/s13019-024-02631-z (PMC10953136; doi:10.1186/s13019-024-02631-z)
Supplement: Supplementary file 1 — Additional file 1: Supplementary Figure 1. Combined papillary muscle approximation and restrictive mitral annuloplasty. Supplementary Figure 2. The anatomy of the PMs (papillary muscles) is the key to surgical correction. [file 13019_2024_2631_MOESM1_ESM.docx]

**Supplementary Material**

**1. Intervention**

The surgery involved PMA along with RMA and was conducted through median sternotomy under normothermic cardiopulmonary bypass conditions using intermittent anterograde blood cardioplegia. Before beginning with the cardiopulmonary bypass, transoesophageal echocardiography was carried out to confirm that there were no MV structural abnormalities, and all eligibility criteria were met.

End-diastolic and end-systolic distances between the interpapillary muscles were compared to preoperative values measured through transthoracic echocardiography in the parasternal short-axis view of the LV. Comparing IPMD in physiological and pathological conditions is crucial for procedure success. In normal physiological conditions, the distance between the interpapillary muscles during mid-systole ranges from 12.9 mm to 22.5 mm. It should be noted that these measurements have variations when adjusted for BSA (body surface area) and age correction. The IPMD measurement, when adjusted for BSA and age, is 10.5 mm with a margin of error of 3.3 mm.^1^  Preoperative values for RMA group are ED IPD, mm 44.2 +/- 3.9; MS IPD, mm 39.1 +/- 6.1 while for PMA group are ED IPD 44.7 +/- 4.2 and MS IPD, 40.5 +/- 4.6, respectively.

To obtain the best view of the MV through a left atriotomy, we utilized the Carpentier retractor (Tisurg, Jiangsu, People’s Republic of China) for support. During surgical examination, all patients displayed lesions commonly associated with Carpentier type IIIb classification, which confirmed the echocardiographic diagnosis. The MV geometrical disorder was identified by changes in three metrics: annular dilation in the anteroposterior axis, tenting area, and interpapillary muscle distance. In all patients, we observed restriction of the leaflets due to excessive traction, resulting in a lack of coaptation.

After performing diastolic cardioplegic arrest, the first step was to carefully identify and inspect the papillary muscles intraoperatively. To confirm the echocardiographic findings, the inter-papillary muscle distance (IPMD) was measured using a flax thread. The PM distance was then decreased, and a second measurement was taken through the same method to confirm the results. It should be noted that papillary muscles identified anatomically as type I and II were attached using a CV-4 Gore-Tex suture (W. L. Gore & Associates, Flagstaff, Ariz). The stitch was positioned on the head of every PM (see Figure 1 up). For type III, IV, or V PMs, a 4-mm Gore-Tex tube (W. L. Gore & Associates) was used to encircle the bodies of the posteromedial and anterolateral PMs, which were then reinforced together (see Figure 1 up). The identification of two separate heads allowed for the simultaneous approximation of both posteromedial PMs, reducing MV tenting.

Special attention was given to assessing the arrangement of chordae to improve regurgitation correction, involving a thorough evaluation of the precise anatomical location of chordae tendinea. Typically, chordae originating from the PPM are inserted on the scallop P2 and P3 of the posterior leaflet, while those stemming from APM are destined for the anterior leaflet, primarily contributing to the development of the 'seagull sign' and corresponding tenting.

The next step involved calibrating the anterior leaflet area to carry out the RMA procedure. Subsequently, the AL was precisely measured with a prosthetic ring obturator. The annuloplasty was performed with a Carpentier-Edwards Physio ring (Edwards Lifesciences, Irving, Calif), which was undersized by 2 sizes. Afterwards, the prosthetic ring was secured with 2/0 braided sutures placed 1mm from the leaflet's hinge on the atrial wall. Sutures were placed in a counterclockwise direction, beginning from the posterior commissural area. To enhance the downsizing effect at this level, bigger bites were taken on the posterior part of the annulus, from trigone to trigone. Afterward, all sutures were threaded through the prosthetic ring cuff and tied together once the ring was lowered into position.

The third phase involved performing a concomitant CABG operation in all patients. For complete revascularisation of diseased coronary arteries, we utilised a single or double ITA. In patients under 65 years of age, we preferred using both Y-shaped and in situ RITA and LITA.

**2. Technical consideration of intervention.**

**2.1. Papillary Muscle Anatomy and Vascularization**

We have identified five different segmentation and morphological types of papillary muscle in the mitral valve. The most common types are: type I, which consists of a single uniform unit; type II, which features a groove with two apexes; type III, which has fenestrations with muscular bridges; type IV, where there is complete separation in two adjacent heads; and type V, where there is complete separation with two distant heads (see Figure 2A). An adequate analysis of papillary muscle handling requires an understanding of its division occurring in two directions, corresponding to the sagittal and coronal planes or solely the coronal plane (refer to Figure 2 B).^2-5^ We observed scarce vascularization of PPM among individuals with an ischemic mitral prolapse, with the prevalence ranging at around 91%. This is attributable to the various coronary vascular distributions in the papillary muscle, resulting in an uneven blood supply distribution. This phenomenon explains the infrequent involvement of the anterior PM and the entire segment of the ventricular wall. The perfusion of this muscle is assured by both the left anterior descending coronary artery and the diagonal branch. Biomechanically, evidence suggests that the chordae exert tension on the APM, which leads to relatively low dynamic stress-strain due to its surface location with regard to the annulus. Conversely, the disparity observed in PPM injury can be attributed to its heightened susceptibility to ischemia (noted in 91% of our studies), owing to its perfusion by either the right coronary or circumflex artery.^2^ Additionally, the increased stress experienced by PPM stems from its location deep within the left ventricle, resulting in augmented shear force.

Analysis of PM microcirculation reveals an independent blood supply ensured by a segmented distribution and a well-defined arterial trunk, known as Kugel’s artery, which perforates the PM from base to apex. This discovery regarding microcirculation and the anatomical characteristic of the PM highlights the relative importance of one of the two circulatory systems. The resilience of the papillary muscle depends on its morphology and location within the ventricle, as well as the presence of muscular bridging that encourages collateralization. The importance of the truncal system is further amplified when the papillary muscle is more separate from the ventricular wall, as seen in type IV-V. Such patterns increase the vulnerability of the apex to rupture, caused by the fragile supply of blood to the truncal region, and the amount of physical strain.^2,3^

**2.2. Surgical consideration related to the technique.**

The aim during the surgical procedure was to reduce the end-diastolic interpapillary distance by an average of 30% compared to the pre-surgical measurement (using the flax thread method). This was confirmed via intraoperative transoesophageal echocardiography. The degree of reduction achieved resulted in local left ventricular remodeling. The increased proximity of the papillary muscles can be observed through the overall positive recovery of left ventricular remodeling. The reduction in IPMD can be attributed to the positive effects of reverse remodeling of the left ventricle.

During the reapproximation procedure, it is important to consider several factors. Firstly, the echography quantification of MR should be achieved in endsystole. This involves measuring MR during endsystole. Secondly, the management of the papillary muscle should be carried out during the surgical ventricular diastole, where IPMD is closely associated with the expansion of the left ventricular chamber. When measuring mitral regurgitation, the IPMD measurement is higher in end-diastole than in end-systole. Additionally, reducing the LV size results in an additional IPMD approach.

Some authors have considered relocating PMs in SIMR patients^6,7^. However, we have decided against this approach because it fails to address the multidirectional displacement and migration of the PM, potentially causing increased tension at the posterior trigone level and negative biomechanical effects. Indeed, Watanabe discovered that relocating the PPM may have a restrictive impact on the MV if directed solely to the posterior leaflet.

This relocation induces a tilting effect on the posterior annulus, thus increasing posterior tethering. In these instances, the V form of the PPM, which identifies two distinct heads for the PM and relative chordae for both leaflets, becomes extremely important. Therefore, the relocation of both PMs is absolutely necessary.

**Supplementary Figure 1.** Papillary muscle approximation, in combination with restrictive annuloplasty undersizing, demonstrated advantages in ventricular remodeling and reduced regurgitation recurrence during follow-up, when compared to restrictive annuloplasty alone. *Nappi, F. et al. J Am Coll Cardiol. 2016;67(20):2334–46.*

**

1. **Supplementary Figure 2.** The papillary muscle of the mitral valve is typically composed of five distinct segmentation and morphological types. The most common patterns are illustrated in **Figure UP**, including type I, a single uniform unit; type II, a groove with two apexes; type III, fenestrations with muscular bridges; type IV, complete separation in two adjacent heads; and type V, complete separation with two distant heads. To analyze papillary damage adequately, it is necessary to consider the division that can occur in two directions: either along a sagittal plane, as shown in **Figure UP**, or along a coronal plane, as shown in **Figure Down**. The anterior leaflet is denoted by A, the commissure by C, and the posterior leaflet by P. *From Nappi F et al Int J Cardiol. 2016 Feb 1 ;204 :218-28.*

**Supplementary References**

1. Sonne C, Sugeng L, Watanabe N, Weinert L, Saito K, Tsukiji M, Yoshida K, Takeuchi M, Mor-Avi V, Lang RM. Age and body surface area dependency of mitral valve and papillary apparatus parameters: assessment by real-time three-dimensional echocardiography. Eur J Echocardiogr. 2009 Mar;10(2):287-94.
2. Nappi F, Nenna A, Spadaccio C, Lusini M, Chello M, Fraldi M, Acar C.. Predictive factors of long-term results following valve repair in ischemic mitral valve prolapse. Int J Cardiol. 2016 Feb 1 ;204 :218-28.
3. Nappi F, Cristiano S, Nenna A, Chello M. Ischemic mitral valve prolapse. J Thorac Dis. 2016 Dec;8(12):3752-3761.
4. Nappi F, Spadaccio C. Obstructive Cardiomyopathy and Tethering in Ischemic Mitral Regurgitation: Two Sides of the Coin. Ann Thorac Surg. 2019 Jun ;107(6) :1911-1912
5. Jouan J, Tapia M, C Cook R, Lansac E, Acar C. Ischemic mitral valve prolapse: mechanisms and implications for valve repair. Eur J Cardiothorac Surg. 2004 Dec;26(6):1112-7.
6. Watanabe T, Arai H, Nagaoka E, Oi K, Hachimaru T, Kuroki H, et al. Influence of procedural differences on mitral valve configuration after surgical repair for functional mitral regurgitation: in which direction should the papillary muscle be relocated? J Cardiothorac Surg. 2014 ;9 :185.
7. Fattouch K, Castrovinci S, Murana G, Dioguardi P, Guccione F, Nasso G, et al. Papillary muscle relocation and mitral annuloplasty in ischemic mitral valve regurgitation: midterm results. J Thorac Cardiovasc Surg. 2014 ;148 :1947-503.
